# Mapping the D.melanogaster En1A Enhancer Modules Responsible for Transcription Activation and Long-Distance Enhancer-Promoter Interactions

**Published:** 2017

**Authors:** L. S. Melnikova, E. A. Pomerantseva, V.V. Molodina, P. G. Georgiev

**Affiliations:** Institute of Gene Biology, Russian Academy of Sciences, Vavilova str. 34/5, Moscow, 119334, Russia

**Keywords:** Drosophila melanogaster, long-distance interactions, transcription activation, enhancer structure, yellow gene

## Abstract

The structure of the new enhancer En1A of the 1A region of the X chromosome of *D. melanogaster *was investigated. Two distinct regulatory elements were found. The first element is responsible for transcription activation, and the second element provides specific interaction with the promoter of the *yellow *gene. The findings support the hypothesis of a modular structure for enhancers, including certain sequences that bind transcription activators and special communication elements providing long-distance enhancer-promoter interaction.

## INTRODUCTION

One of the key properties of enhancers is their ability to specifically activate the transcription of the target gene that, in some cases, covers a distance of tens or even hundreds of kilobase pairs [1]. However, the mechanisms that are at play for maintaining specific long-distance interactions between enhancers and promoters remain elusive. In some cases, the *cis-*regulatory sequences found within the promoter regions of eukaryotic genes have been known to enable communication between an enhancer and a promoter [2-4]. Collected data [5] suggest that the specificity of some enhancers is due to the presence in them of binding sites for the said transcription factors (TF), which are responsible for transcription activation, and of proteins providing a stable long-distance enhancer-promoter interaction.

The aim of the current study was to investigate the new enhancer En1A found in the intron of the unexplored gene *CG3777 *located on the X chromosome.

The En1A enhancer was shown to have a modular structure. We found the activation and communication elements in the structure of En1A. The activation element is able to functionally replace the *yellow *gene body and wings enhancers; i.e., stimulate transcription in the corresponding cuticular structures. The communication element is necessary for the interaction between En1A and the *yellow *gene promoter and able to provide long-distance GAL4-dependent transcription activation.

## EXPERIMENTAL PROCEDURES

All constructs are based on a pCaSpeR3 vector containing the *mini-white *gene. The plasmid vector pCΔ derived from pCaSpeR3, which contains a deletion of the *mini-white *gene, has been described previously [6].

For the constructs EcoRI–PstI-Y, PstI–PvuII-Y, and HindIII–^y+s^-Y, the corresponding restriction fragments of the chimeric element from the *y+s *allele were used. The fragments were inserted upstream of the *yellow *gene promoter at position -343 bp (hereinafter, including figures, the numeration within the *yellow *locus is determined relative to the gene transcription initiation site) at the KpnI restriction site.

For (a1–a2)Y construct design, *yellow *cDNA lacking a bristle intron and an enhancer was used (pCaSpeR3-Yil). A fragment of 362 bp was amplified from the genomic DNA of a *y+s *fly line using the primers a1 (5’-CTTTTTGCATACACATCCAC-3’) and a2 (5’-GCTGATGGAAGTTGCAGA-3’) and cloned into a vector based on the pBlueScript plasmid between two *loxP *sites at the EcoRV site (a1–a2/lox). Next, a a1–a2/ lox fragment was cloned into the vector pCaSpeR3-Yil at the KpnI site at position -343 bp. All of the constructs had a deletion of the *yellow *regulatory sequence of up to -343 bp (XbaI–Eco47III fragment).

In order to obtain a vector lacking *yellow *body and wing enhancers, the XbaI–Eco47III fragment containing body and wing enhancers was deleted from the pCΔ vector, which contains a complete sequence of the *yellow *gene (CΔ-y (-890)). In the constructs YG4(Cm1A), eveYG4(Cm1A), and ΔeveYG4(Cm1A), a DNA fragment containing 10 binding sites for the yeast activator protein GAL4 (two copies of five binding sites from the pUAST plasmid vector) was inserted into a CΔ-y (-890) vector at the 3’-end of the *yellow *gene at the SmaI restriction site, while a a1–a2/lox fragment was inserted at the SacI restriction site. The procedures for the substitution of the -68 … +130 bp sequence in the *yellow *promoter with the sequence of the *eve *promoter and how to obtain pre-promoter -69… -100 bp deletion have been described previously [2].

DNA constructs and a *P-*element with defective inverted *P25.7wc *repeats used as a source of transposase were injected into pre-blastoderm-stage embryos of *yacw1118*. The survived flies were crossed with a *yacw1118 *line. The transgenic flies were selected based on the phenotypic expression of the genes *white *and *yellow*. Lines with a single construct copy in the genome, which was confirmed by Southern blot analysis, were selected for further studies. Details of the cloning of *yellow *gene sequences into vectors, molecular methods of research, embryo transformation and production of transgenic lines of Drosophila, phenotypic analysis of *yellow *gene expression in transgenic lines, induction of site-specific recombination between *loxP *sites, and induction of GAL4-dependent activation in transgenic lines have been described in detail in previous studies [2, 5, 6].

Line *yw^1118^; P[w+, tubGAL4]117/TM3, Sb *(Bloomington Center #5138) was used for the induction of the yeast protein GAL4. Line *y ac w^1118^;Cyo, P[w+,cre]/Sco *was used for the induction of recombination between the *loxP *sites. The nucleotide sequence of the gene *CG3777 *and the structure and profile of its expression are presented in the FlyBase database (http://flybase.org/reports/FBgn0024989.html).

## RESULTS AND DISCUSSION

In *Drosophila melanogaster, *gene *yellow *is responsible for the pigmentation of cuticular structures: the body, wings, and bristles. The enhancers that control *yellow *expression in the body and wing cuticle are located on the 5’-end of the gene, whereas the enhancer responsible for expression in bristles is located in the intron [7]. In wild-type flies, the body, wings, and bristles have a dark color.

The allele *y^2^*is often used as a model system in works that study transcriptional regulation in *D. melanogaster*. The *y^2^* allele has an incorporated retrotransposon, MDG4 (*gypsy*), between the promoter and enhancer of the body and wings of the *yellow *gene [8]. As a result, a Su(Hw) insulator comprising MDG4 blocks *yellow *activation through body and wing enhancers. Thus, the *y2 *phenotype is characterized by a yellow color of the body and wings, while the bristles are dark-colored.

**Fig. 1:**
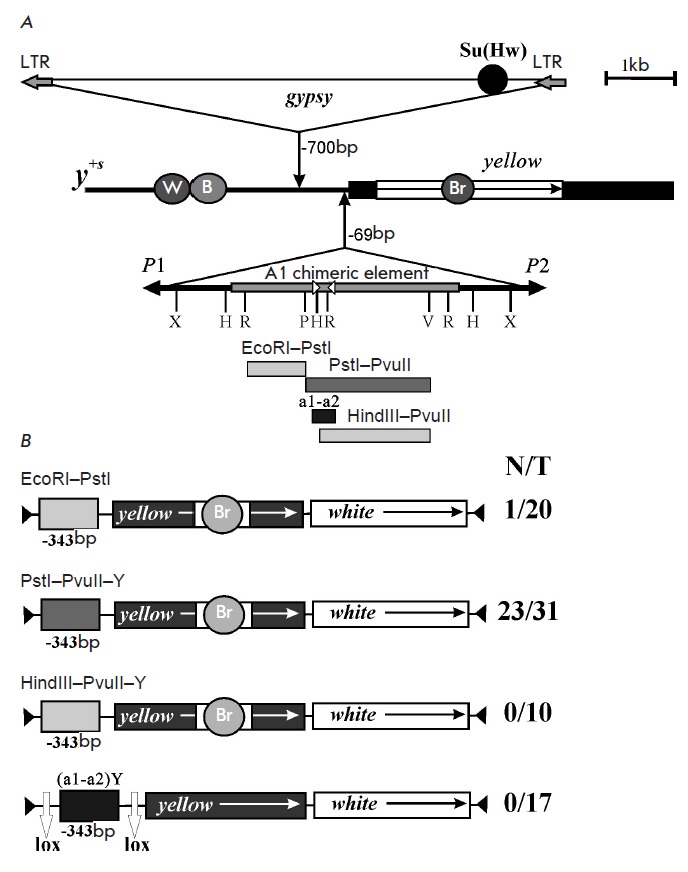
Mapping of a new enhancer, En1A, of the 1A region of the X chromosome. *A *– schematic representation of the structures of the *yellow *locus and *y^+s^*allele. The *Yellow *exons and intron are depicted by black and white boxes, respectively. The direction of transcription is indicated by an arrow. Grey circles represent the tissue-specific transcriptional enhancers responsible for *yellow *expression in the wings (W), body (B), and bristles (Br). Triangles indicate insertions of the *gypsy *retrotransposon and chimeric element. Long terminal repeats (LTR) at the ends of the retrotransposon are shown by gray arrows. Black arrows in the chimeric element indicate the size and orientation of *P*-element sequences. The internal region of the chimeric element corresponding to the sequence of gene *CG3777 *is depicted by a gray rectangle. Abbreviations of the restriction sites are: R – EcoRI; X – XhoI; P – PstI; H – HindIII; V – PvuII. Localization and direction of PCR primers are shown by open triangles. The rectangles under the scheme of the chimeric element correspond to fragments comprising transgenic constructs. *B *– schematic representation of transgenic constructs including fragments of the chimeric element. Arrows indicate the direction of *yellow *and *white *transcription. Ends of the *P*-element in the vector are shown by black triangles. White vertical arrows signed “*lox*” indicate *Cre *recombinase binding sites. N is the number of fly lines with a dark body and wings pigmentation. T is the total number of transgenic lines.

The superunstable allele *y^+s^* (*Fig. 1A*) was obtained by induction of P-M hybrid dysgenesis in a line containing the *y^2^*mutation [9]. Allele derivatives *y^+s^*– *y^2s1^*and *y^2s2^*containing a chromosome X region 1A duplication in the pre-promoter region of *yellow *have also been obtained [9, 10]. The study of the structure of the alleles *y^2s1^*and *y^2s2^*made it possible to identify the regulatory element 1A-RE, which activates long-distance *yellow *expression and is a *yellow*-specific insulator, within the duplicated fragment comprising the region 1A [10]. In the presented paper, we continued our thorough study of the structure of the *y^+s^*allele.

The mutation *y^+s^*was a result of the introduction of a chimeric 5,4 kb element at position -69 bp with simultaneous deletion of the *yellow *sequence between -146 and -70 bp. The chimeric element consists of 1,2 kb *P*-elements located “tail to tail” and a 3 030 bp sequence trapped between them, which presents a duplication of the region 1A of chromosome X and is located distal to the *yellow *locus in the genome (*Fig. 1A*) [9]. This duplication includes a fragment of the unexplored gene *CG3777*, which is expressed at the same stages of development as the *yellow *gene.

The body and wings of the flies carrying allele *y+s *exhibit a dark color close in intensity to the color of wild-type flies. Hence, gene expression is activated in the body and wings in the case when the 1A region from chromosome X is shifted to the *yellow *gene, despite the fact that the Su(Hw) insulator blocks the corresponding enhancers. We managed to localize a 1748 bp enhancer, which was called enhancer 1A (En1A), in the relocated DNA sequence by using transgenic constructs (*Fig. 1B*).

First, we tested two restriction fragments which together cover most of the region 1A duplications: EcoRI–PstI of 771 bp and PstI–PvuII of 1748 bp (*Fig. 1A*). In the transgenic constructs EcoRI–PstI-Y and PstI– PvuII-Y, these fragments were located upstream of the *yellow *promoter at position -343 bp (*Fig. 1B*). Both constructs contained no body or wing enhancers. Among the lines carrying the EcoRI–PstI-Y construct, 19 flies out of 20 had an uncolored body and wings. The phenotype of the flies from transgenic PstI-PvuII-Y lines was similar to the wild-type phenotype in 23 out of 31 lines obtained, which is proof of the ability of the 1748 bp fragment to functionally replace the body and wing enhancers of the *yellow *gene. Hence, the En1A enhancer is localized within the PstI–PvuII region.

In order to accurately map En1A, two genetic constructs containing distinct PstI–PvuII fragments incorporated at position -343 bp were designed: HindIII– PvuII-Y and (a1–a2)Y (*Fig. 1B*). The HindIII–PvuII fragment, of 1 511 bp (*Fig. 1A*), had no enhancer properties: the body and wings of the flies were yellow in all 10 transgenic HindIII-PvuII-Y lines (*Fig. 1B*). A bioinformatic analysis of the structure of the PstI–PvuII sequence revealed a 362-bp fragment comprising recurring motifs, which, possibly, could serve as binding sites for regulatory proteins. This DNA fragment was amplified by PCR using the primers a1 and a2 and then incorporated upstream of the *yellow *promoter as part of the (a1–a2)Y construct (*Fig. 1B*). Fragment a1–a2 is surrounded by the *Cre *recombinase recognition sites (*loxP *sites), which allow *in vivo *excision of the analyzed element [11]. It should be noted that *yellow *cDNA contained no bristle enhancer in the construct (a1–a2)Y (*Fig. 1B*). We obtained 17 transgenic lines carrying the construct. Despite the absence of a bristle enhancer, the flies of all the lines had the y^2^ phenotype. Excision of the a1–a2 sequence resulted in the disappearance of pigmentation in bristles in 12 out of 15 lines. Thus, the studied 362 bp fragment within the (a1–a2)Y construct interacted with a promoter and stimulated *yellow *expression in bristles but was incapable of functionally substituting body and wing enhancers.

The obtained results allowed us to suggest that enhancer En1A has a heterogeneous structure. One part of the enhancer, (a1–a2) of 362 bp, named the “communication part” (hereinafter Cm1A), alone stimulates *yellow *expression only in the bristles. However, it is necessary for the stimulation of *yellow *expression by the full-length En1A in the body and wings. Another part of the PstI–PvuII sequence of 1,386 bp is capable of inducing a high level of *yellow *expression in the body and wings only in combination with the communication part (*Fig. 1 A,B*). Full-length En1A of 1,748 bp activates *yellow *transcription in all cuticular structures. Apparently, the 1,386-bp fragment contains binding sites for *yellow *transcription activators in the body and wings, but their interaction with the promoter is provided by Cm1A-binding proteins.

**Fig. 2:**
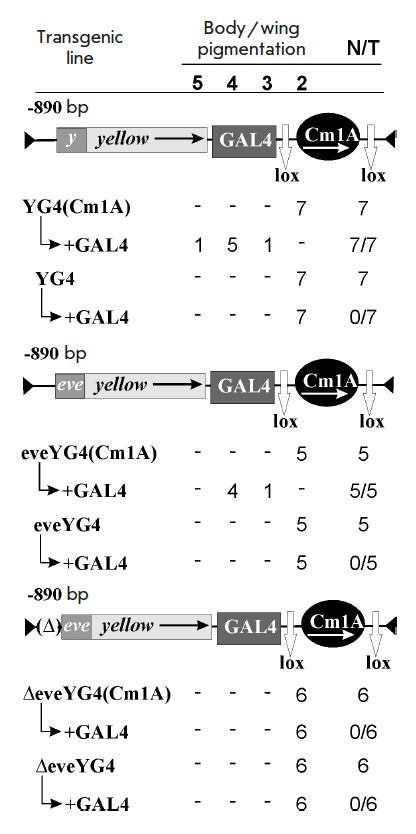
Screening of the communication properties of the Cm1A fragment. Results of the phenotypic analysis of the flies in transgenic lines are presented under construct schemes. Designations: *y *– *yellow *gene promoter; *eve *– *even skipped *gene promoter; black ellipse – Cm1A communicator. The arrow inside an ellipse indicates the transcription direction. Deletion of the *yellow *gene sequence of -69 to -100 bp (TE) is marked by Δ. Pigmentation of the body and wings is numbered from 5 (dark color, as in the wild-type) to 2 (yellow color corresponding to the phenotype of the *y^2^*allele). The designation “+ GAL4” refers to the derivatives obtained after GAL4 activation in transgenic lines of the corresponding genotype. N is the number of lines of flies that acquired a new phenotype after Cm1A deletion or by crossing with the line expressing GAL4. T is the total number of lines examined for each particular construct. For other designations, see *Fig. 1*.

To further explore the communication properties of the Cm1A element, we used a model system based on the properties of the yeast activator GAL4. This activator is known to stimulate promoters of various genes in the Drosophila genome [2]. However, GAL4, located at the 3’-end of the gene, is incapable of transcription activation [12]. In the construct YG4(Cm1A), the protein GAL4 binding sites and a potential communicator, Cm1A, surrounded by *loxP *sites were incorporated at the 3’-end of the *yellow *gene. In addition, the 5’ sequence of *yellow *containing body and wing enhancers (up to -890 bp) was deleted (*Fig. 2*). In seven transgenic lines carrying the YG4(Cm1A) construct, the flies had a y2 phenotype. Thus, in the absence of GAL4 activation, the Cm1A fragment is incapable of activating the transcription of *yellow *in the body and wings. Then, we crossed YG4(Cm1A) transgenic lines with a line expressing the GAL4 protein. As a result of GAL4 activation, the body and wings of the flies in all the lines acquired a darker color (*Fig. 2*). Deletion of Cm1A led to a decrease in *yellow *expression to its initial level. Therefore, the Cm1A element, indeed, has communication properties. It provides stable long-distance interaction with the GAL4 activator and *yellow *promoter.

**Fig. 3:**
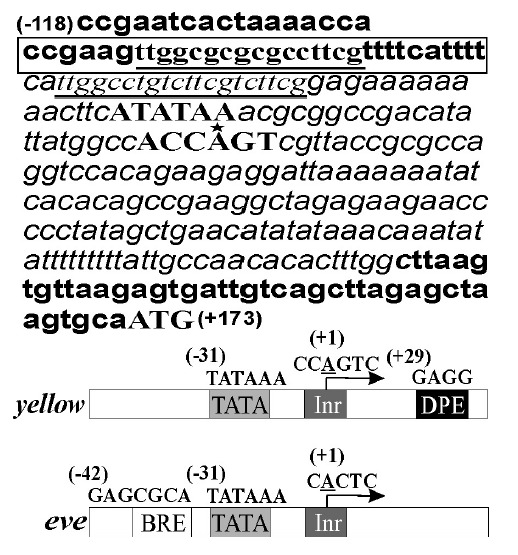
Promoter regions of the *yellow *and *eve *genes. The upper part of the figure depicts the sequence of the *yellow *promoter region. The TATA promoter, initiator, and translation start site are designated by capital letters. The 198-bp *yellow *region substituted in the eveYG4(Cm1A) and ΔeveYG4(Cm1A) constructs is shown in italic. The transcription initiation site is indicated by an asterisk. Partially duplicated sequences (a putative tethering element) upstream of TATA are underlined. The sequence of -69 to -100 bp including TE of the *yellow *gene is boxed. Figures in parentheses indicate the relative distance from the transcription start site. The core promoter elements of the *yellow *and *eve *promoters are shown schematically. Arrows indicate the transcription direction. Previously reported sequences of the TATA box (TATA), initiator (Inr), downstream promoter element (DPE), and the putative sequence of the TFIIB binding element (BRE) are shown. The sequences of core promoter elements are designated by capital letters.

Earlier, we had localized TE at -69 … -100 of *yellow, *which provides long-distance interaction of body and wing enhancers with the *yellow *promoter, as well as the heterologous promoter of the gene *eve *[2]. We hypothesized that the Cm1A communicator functionally interacts with TE of the *yellow *gene. In order to test this hypothesis, the constructs eveYG4(Cm1A) and ΔeveYG4(Cm1A) were obtained (*Fig. 2*). In both constructs, the *yellow *gene promoter was replaced by a heterologous promoter of the *eve *gene (-68 … +130 bp) (*Fig. 3*). Moreover, the TE sequence of the *yellow *gene was deleted in the ΔeveYG4(Cm1A) construct (*Fig. 2*, (*Fig. 3*). The results obtained during a phenotypic analysis of five eveYG4(Cm1A) transgenic lines were similar to the results of a YG4(Cm1A) line analysis. As in the previous case, the communicator provided GAL4-dependent transcription activation of *yellow*. Since the structures of the *yellow *and *eve *promoters are different (*Fig. 3*), one can assume that the core elements of the promoter are not involved in the functional interaction with Cm1A. In six transgenic lines carrying the ΔeveYG4(Cm1A) construct, GAL4 activation did not lead to changes in the initial y2 phenotype (*Fig. 2*). Hence, the communicator Cm1A is incapable of supporting long-distance interaction between the transcription activator and the promoter of the gene in the absence of *yellow *gene TE. Apparently, the proteins binding the communication element Cm1A can interact with the proteins recruited to TE of the *yellow *gene. Such interaction brings the GAL4 activator and promoter spatially together in the described model system, which enables contact between the activation complex recruited to the GAL4 sequences and the transcriptional complex of the promoter.

## CONCLUSION

The presented data allow us to conclude that the new enhancer En1A has a modular structure. In a previous study, we showed that the regulatory system of *white *also includes elements that do not affect transcription but provide long-distance enhancer-promoter interaction. The pre-promotor region and the eye enhancer of gene *white *contain binding sites for the Zeste protein. The Zeste protein is not involved in transcription activation but allows the *eye *enhancer to activate a long-distance promoter through binding to its target sites [5]. The results of the current study support the hypothesis that the regulatory regions of various genes have a modular structure and include activation elements that bind to transcription factors, initiating and providing efficient transcription and communication elements that bind proteins, providing spatial contact between an enhancer and a promoter. The described model systems can be used to study the enhancer structure and identification of the sequences involved in long-distance interactions between the regulatory elements of the genome.

## Abbreviations

TF: transcription factors
En1A (Enhancer 1A): enhancer of the 1A region of the X chromosome;
Cm1A (Communicator 1A): communication element of the En1A enhancer
TE (tethering element): a regulatory element in the promoter responsible for long-distance enhancer-promoter interactions
